# Prevalence and risk factors of pneumococcal nasopharyngeal carriage in healthy children attending kindergarten, in district of Arsi Zone, South East, Ethiopia

**DOI:** 10.1186/s13104-019-4283-3

**Published:** 2019-05-07

**Authors:** Gizaw Abaye, Hailu Fekadu, Kelili Haji, Desalegn Alemu, Antehun Alemayehu Anjulo, Debela T. Yadate

**Affiliations:** 1 Department of Medical Laboratory Science, College of Health Sciences, Arsi University, Asella, Ethiopia; 2 Department of Public Health, College of Health Sciences, Arsi University, Asella, Ethiopia; 3 Department of Anatomy, College of Health Sciences, Arsi University, Asella, Ethiopia; 4 Department of Medical Laboratory, College of Health Sciences and Medicine, Wolaita Sodo University, Wolaita Sodo, Ethiopia; 5 Department of Physiology, College of Health Sciences, Arsi University, Asella, Ethiopia

**Keywords:** Nasopharyngeal carriage, *S. pneumoniae*, Antimicrobial susceptibility pattern, Risk factors

## Abstract

**Objective:**

*S. pneumoniae* responsible for a range of respiratory infections from uncomplicated to severe invasive pneumococcal disease. Nasopharyngeal specimens were collected from children attending kindergarten and aged ≤ 6 years from February, 2017 to June, 2017 to assess the nasopharyngeal carriage and antimicrobial susceptibility pattern of *S. pneumoniae*. Parents of children interviewed using questionnaire and check list to identify associated factors. An antimicrobial susceptibility test performed using disk diffusion method.

**Results:**

Overall pneumococcal carriage were 18.4% (88/477). No significant variation in colonization based on sex and age of children. Children living with siblings (1–2) < 6 years in household (adjusted odd ratio = 16.06; 95% confidence interval 6.21–41.55) and > 5 person per household (adjusted odd ratio = 3.27; 95% confidence interval 1.50–7.14) were associated with higher *S. pneumoniae* carriage. Non- exclusive breast feeding (adjust odd ratio = 6.00; 95% confidence interval 3.33–10.80) and horse cart transportation (adjusted odd ratio = 2.75; 95% confidence interval 1.05–7.22) increases carriage. *S. pneumoniae* showed 21 (23.9%) resistance to erythromycin, 18 (20.4%) to amoxicillin, 13 (15.0%) to penicillin, and the least 1 (1.1%) to augmentin.

## Introduction

Pneumococcal infection is a leading cause of mortality resulting from infectious disease worldwide. Mortality is highest in patients who develop sepsis or meningitis [1, 2]. Carriage is generally higher in low and middle-income countries and among economically deprived populations. Pneumonia affects children and families everywhere, but is most prevalent in South Asia and Sub-Saharan Africa. The carriage might also vary between low and middle-income countries. Higher prevalence reported in sub-Saharan Africa countries including Gambia, Ethiopia and Mozambique [2–4].

The pneumococcal-conjugate vaccine was introduced in Ethiopia starting from November 2011 across the country. The vaccine is given to under 5 children at 6, 10 and 14 weeks of age along with DPT-HepB-Hib. *S. pneumoniae* remains a major cause of childhood illness and death. It kills at least one million children under the age of five every year, > 70% of these deaths are in low and middle-income countries. Colonization begins very soon after birth. Most studies have found asymptomatic carriage of between 30 and 62% in children under 2 years of age [5–8]. Studies showed that, pneumococcal colonization in infants starts at 6 months of age. The carriage then increases during the first years of life and reaches a peak in children of 2–3 years of age. The carriage decreases as age increases and low in adults, but in the elderly, the carriage eventually increases [9–11].

Young children considered to be the most important carrier for horizontal dissemination of bacterial strains within the community. Children attending kindergartens are at increased risk for infectious diseases. The nasopharyngeal carriage are double among children attending day care centers outside the home as compare to all children 6 years old or younger [12, 13]. Children attending day-care centers are at higher risk of carriage of *S. pneumoniae* in general and antibiotic-resistant *S. pneumoniae* in particular, than are children who are cared for at home [14].

Like in other low and middle-income countries, children in Ethiopia are seriously affected by communicable disease among which acute respiratory infection plays the major role [15]. This study intended to assess nasopharyngeal carriage of *S. pneumoniae*, antimicrobial susceptibility pattern, and possible risk factors among preschool children in kindergarten in Arsi zoni, Ethiopia. It would be useful to fill gap in terms of limited data and to strengthen the current pneumococcal immunization program and give appropriate information for policy makers.

## Main text

### Methods and materials

#### Study design and participants

Children between 2 and 6 years of age who attend kindergartens in district of Arsi Zone, Ethiopia were recruited from February 2017 to June 2017. Multi-stage sampling technique was used and simple random sampling method was applied for selection of districts and Kindergarten and systematic random sampling was used for selecting 477 study participants. All apparently healthy children’s ≤ 6 years were included.

#### Specimen collection, isolation, and identification

A nasopharyngeal secretion was collected from each child using a sterile synthetic cotton swab on flexible aluminum wire and inoculated onto skim milk, tryptone, glucose, glycerol transport medium within 4 h of collection. The specimens were then seeded onto 5 mg/mL gentamycin supplemented blood agar plates by rolling the swab over a small area of the plate and streaking the sample using a sterile loop. The inoculated media were then incubated at 37 °C for 20–24 h in 2% to 5% CO_2_ rich atmosphere (candle jar method). *S. pneumoniae* identification was based on colony characteristics (α-hemolysis), microscopic morphology by gram’s stain, optochin sensitivity, and bile solubility [16–18].

#### Antimicrobial susceptibility

Disk diffusion method (Kirby–Bauer) was carried out using on Muller Hinton agar supplemented with 5% sheep’s blood [17–20]. To standardize the inoculum density for susceptibility tests, a 0.5 McFarland standard was used. Within 15 min after adjusting the turbidity of the inoculums suspension, a sterile cotton swab was dipped into the adjusted suspension. The dried surface plates were inoculated by streaking the swab over the entire sterile agar surface. The antimicrobial disks were placed on the lawn of bacterial isolates using sterile forceps. Inoculated media were incubated in a 5% CO_2_ atmosphere for 18–24 h at 37 °C. The results were interpreted by comparing the results to the standard zone sizes of the Clinical and Laboratory Standard Institute. *S. pneumoniae* ATCC 49619 was used as the quality control strain for each run as recommended by the Clinical and Laboratory Standard Institute [17–20].

#### Data analysis

Epi info version 7 used to enter data and then, exported to SPSS version 21 for analyses. A descriptive analysis was used to determine the demographic characteristics and the prevalence of each isolate. A bivariate and multivariate logistic regression analysis was done to evaluate the association between the characteristics of the children and the carriage of *S. pneumonia* and also to control for all possible confounding factors. All the variables significantly associated during crude analysis with the p-value ≤ 0.25 were candidate for adjusted analysis. All tests with p value < 0.05 were considered statistically significant.

### Results

#### Demographic characteristics and risk factor analysis

A total of 477 children were enrolled in the study, of whom 244 (51.2%) were boys (mean age = 5.1 and SD = 0.74 years). Three hundred and seventy-one (77.8%) lived in ventilated environments, and 45 (9.4%) households had > 5 family members. The majority of the children 261 (54.7%) were living within a 3–4 room (house with 3–4 room), and only 79 (16.6%) of the children share room with < 2 years children in the household. Children without exclusive breast feeding 76 (16.4%) and horse cart transportation 36 (7.9%) increases carriage (Table 1).

**Table 1:** Sociodemographic characteristics and risk factor analysis for *S. pneumoniae* carriage in 477 study participants recruited from Kindergarten at selected woreda in Arsi Zone, February, 2017 to June, 2017

Variables	*n* (%)	*S. pneumoniae* (*n* = 88)		OR with 95% CI		
		No	Yes	Crude OR (95% CI)	AOR (95% CI)	p
		n (%)	n (%)			
Sex						
Male	244 (51.2)	199 (81.6)	45 (18.4)	1.00 (0.63–1.59)	0.91 (0.54–1.55)	0.738
Female	233 (48.8)	190 (77.3)	53 (22.7)	1	1	
Age (year)						
≤ 3	7 (1.4)	5 (71.4)	2 (28.6)	0.71 (0.13–3.81)	0.82 (0.13–5.30)	0.839
4	74 (15.5)	64 (87.2)	9 (12.2)	2.05 (0.93–4.51)	2.74 (1.12–6.77)	0.029
5	233 (48.5)	189 (82.4)	41 (17.6)	1.33 (0.81–2.19)	1.29 (0.73–2.27)	0.385
6	163 (34.2)	125 (77.9)	36 (22.1)	1	1	
Exclusive breast feeding						
Yes	399 (83.6)	344 (86.2)	55 (13.8)	1	1	
No	78 (16.4)	45 (57.7)	33 (42.3)	4.59 (2.70–7.81)	6.00 (3.33–10.80)	0.000
Living house						
Ventilated	371 (77.8)	327 (88.2)	44 (11.8)	1	1	
Crowded	106 (22.2)	62 (58.5)	44 (41.5)	5.27 (3.20–8.68)	1.77 (0.89–3.54)	0.105
Number of person per house hold						
2–3	240 (50.3)	219 (91.3)	21 (8.7)	1	1	
4–5	192 (40.3)	149 (77.6)	43 (22.4)	11.92 (5.70–24.91)	8.08 (2.94–22.22)	0.000
> 5	45 (9.4)	21 (47.7)	24 (53.3)	3.96 (2.01–7.79)	3.27 (1.50–7.14)	0.003
Sharing the same room with the following children						
None	195 (40.9)	174 (89.2)	21 (10.8)	1	1	
< 2 years	79 (16.6)	59 (74.7)	20 (25.3)	1.3 (0.65–2.60)	0.60 (0.26–1.37)	0.224
2–6 years	85 (17.8)	54 (63.5)	31 (36.5)	0.46 (0.22–0.96)	0.43 (0.19–0.95)	0.036
> 6 years	118 (24.7)	102 (86.4)	16 (13.6)	0.29 (0.14–0.54)	0.43 (0.20–0.95)	0.038
Number of rooms per house hold						
1–2	168 (35.2)	134 (79.8)	34 (20.2)	1	1	
3–4	261 (54.7)	214 (82.0)	47 (18.0)	0.67 (0.28–1.63)	0.58 (0.21–1.62)	0.297
> 5	48 (10.0)	41 (85.4)	7 (14.6)	0.78 (0.33–1.84)	0.78 (0.31–2.00)	0.609
Transportation to KG						
Foot	415 (87.0)	349 (84.1)	66 (15.9)	1	1	
Horse cart	36 (7.5)	25 (69.4)	11 (30.6)	3.88 (1.71–8.82)	2.75 (1.05–7.22)	0.040
Vehicle	25 (5.5)	14 (66.0)	11 (44.0)	0.34 (0.58–4.78)	1.39 (0.42–4.59)	0.591
Recent antibiotic use (at least one antibiotics)						
None	394 (82.6)	323 (82.0)	71 (18.0)	1	1	
Within 2–4 weeks	18 (3.8)	17 (94.4)	1 (5.6)	3.74 (0.49–28.54)	7.79 (0.82–74.38)	0.075
Within 1–6 months	43 (9.0)	33 (76.7)	10 (23.3)	0.73 (0.34–1.54)	0.90 (0.40–2.87)	0.817
Within 6–12 months	22 (4.6)	16 (72.7)	6 (27.3)	0.92 (0.40–2.87)		0.887
Number of siblings ≤ 6 years in household						
0	311 (65.2)	275 (88.4)	36 (11.6)	1	1	
1–2	140 (29.4)	106 (75.7)	34 (24.3)	17.19 (6.97–42.38)	16.06 (6.21–41.55)	0.000
≥ 3	26 (5.5)	8 (30.8)	18 (69.2)	7.02 (2.80–17.57)	6.58 (2.52–17.16)	0.000
URTIs in the last 3 months one or more episode						
None	398 (83.4)	337 (84.7)	61 (15.3)	1	1	
Tonsillopharyngitis	51 (10.7)	32 (62.7)	19 (37.3)	1.38 (0.29–6.66)	1.36 (0.26–7.21)	0.717
Sinusitis	18 (3.8)	12 (76.7)	6 (33.3)	0.42 (0.08–2.19)	0.47 (0.08–2.61)	0.385
Otitis media	10 (2.1)	8 (80.0)	2 (20.0)	0.50 (0.08–2.19)	0.82 (0.11–6.09)	0.846
LRTIs in the last 3 months one or more episode						
None	452 (94.7)	369 (81.6)	83 (18.4)	1	1	
Bronchitis	11 (2.3)	9 (81.9)	2 (18.1)	1.21 (0.33–4.44)	1.50 (0.32–7.04)	0.609
Pneumonia	14 (2.9)	11 (78.6)	3 (21.4)	1.23 (0.17–9.02)	3.43 (0.30–39.57)	0.323
Previous hospitalization						
Yes	43 (9.0)	17 (39.5)	26 (60.5)	0.11 (0.06–0.21)	0.10 (0.05–0.21)	0.000
No	434 (90.9)	372 (85.7)	62 (14.3)	1	1	
Passive exposure to cigarette smoking						
Yes	28 (5.9)	16 (57.1)	12 (42.9)	0.27 (0.12–0.60)	0.26 (0.10–0.67)	0.005
No	449 (94.2)	373 (83.1)	76 (16.9)	1	1	

*LRTIs* lower respiratory tract infection, *OR* odd ratio, *CI* confidence interval, *URTIs* upper respiratory tract infection, *AOR* adjusted odd ratio, *KG* kindergarten

Of the 477 children, 88 (18.4%) were carriers of *S. pneumoniae*. The highest *S. pneumoniae* carriage was observed in children aged of ≤ 3 years (28.6%). Higher carriage in male 45 (18.4%) than female 53 (22.7%). In children without exclusive breast feeding carriage was 33 (42.3) and 43 (22.4%) and 24 (53.3%) children with *S. pneumoniae* were lived in 4–5 and > 5 number of person per house hold. Children living with 1–2 and ≥ 3 number of siblings ≤ 6 years in household were 34 (24.3%) and 18 (69.2%) carriage, respectively (Table 1).

The highest *S. pneumoniae* carriage (26.8%) was observed in children aged ≤ 3 years old. Though, being within any age groups was no significance associated with *S. pneumoniae* carriage. But, *S. pneumoniae* carriage was significant in children without exclusive breast feeding carriage (AOR = 6.00; 95% CI 3.33–10.80; p = 0.0001). In addition*, S. pneumoniae* carriage was significantly higher in children living with person 4–5 and > 5 per house hold (AOR = 8.08; 95% CI 2.94–22.22; p = 0.0001, and AOR = 3.27; 95% CI 1.50–7.14; p = 0.003, respectively). Moreover, there was a significant association between carriage by *S. pneumoniae* and number of siblings (1–2 and ≥ 3) < 6 years living with children in household (AOR = 16.06; 95% CI (6.21–41.55; p = 0.0001 and adjusted OR = 6.58; 95% CI 2.52–17.16; p = 0.0001, respectively) and being transported to horse cart to kindergarten (AOR = 2.75; 95% CI 1.05–7.22; p = 0.040) (Table 1).

#### Antimicrobial susceptibility patterns of bacterial isolates

Thirteen isolate *S. pneumonia* were susceptible to all of the antibiotics tested, 44 (50.0%) were resistant to one antimicrobial agent, 11 (12.5%) were resistant to two, and 7 (8.0%) to more than two. Thirteen (15.0%) penicillin-resistant *S. pneumoniae* were isolated. Twenty-one (23.9%) *S. pneumoniae* isolates were resistant to erythromycin, and 2 (2.3%) were resistant and 86 (97.7%) were susceptible to vancomycin. All penicillin resistant *S. pneumoniae* isolates were resistant to two or more antimicrobial agents. No antibiotics were susceptible for all isolate (Table 2).

**Table 2:** Antimicrobial susceptibility patterns of *S. pneumoniae*, isolated from 477 study participants recruited from Kindergarten at selected woreda in Arsi Zone, February, 2017 to June, 2017

Antimicrobials	*S. pneumoniae* (n = 88)		
	Susceptible	Intermediate	Resistant
	n (%)	n (%)	n (%)
Amoxicillin	68 (77.3)	2 (2.3)	18 (20.4)
Augmentin	87 (98.9)	0 (00)	1 (1.1)
Ciprofloxacin	78 (88.6)	7 (8.0)	3 (3.4)
Chloramphenicol	83 (94.3)	1 (1.1)	4 (4.5)
Erythromycin	67 (76.1)	0 (00)	21 (23.9)
Penicillin	70 (79.5)	4 (4.5)	13 (15.0)
Vancomycin	86 (97.7)	0 (00)	2 (2.3)

## Discussion

This study describes nasopharyngeal carriage of pneumococcus among preschool children in kindergarten in Arsi zoni, Ethiopia. Studies like this are important for situating burden of disease and for explaining the need for vaccination. According to this study, *S. pneumoniae* carriage was high (18.4%) among children in day care center (DCC). Although nasopharyngeal carriage varies throughout the world, our results are consistent with the findings of a various studies carried out elsewhere [21, 22]. However, this finding was low as compared to the study reported in Northern Spain in healthy children at DCC (89.5%) [8], in Gambian infants (51%) [13] and in Peruvian children (75.3%) [23]. This might be due to living conditions, season, respiratory illness, methodological differences and genetic traits [13, 23].

In our study, age appeared to be positively associated with *S. pneumoniae* carriage. This finding is concordant with previous studies [4, 17, 24]. Children’s at 4 years of age were more colonized than children than other age groups 9 (12.2%). The drop in *S. pneumonia* carriage associated with rising age may reflect the gradual acquisition of mucosal immunity and reduction of exposure. Sharing the same room with 2–6 year old also observed to be risk factor in this study (36.5%). Owning to the close interactions between children and kindergartens, it has been proposed that pneumococci are more efficiently transmitted from the nasopharynx of one child to another in DCCs [25]. In addition, number of siblings 1–2 and ≥ 3 years in household (24.3%) and (69.2%) also identified as risk factor for carriage. Children’s having young siblings had been reported as important risk factors for carriage in children [26]. In this study, earlier antibiotics use, sex, living house, number of rooms per house hold, history of LRTI and URTI were not associated with carriage *S. pneumonia*.

Results of our study revealed quite higher *S. pneumoniae* resistance (20.4%) to amoxicillin; (23.9%) to erythromycin; (15.0%) to penicillin; and (4.5%) to chloramphenicol. However, (20.4%) isolated *S. pneumoniae* were multidrug resistant. Penicillin resistance was consistent with previous studies [13, 17, 27–29] and relatively higher frequencies of penicillin resistance pneumococci among children were also reported [20, 29–31]. A wide geographical variation in the antimicrobial susceptibility of *S. pneumoniae* can be explained by the general consumption of antimicrobial agents. According to our results, *S. pneumonia* susceptibility to augmentin and vancomycin was also high (98.9 and 97.7%, respectively). It was consistent with the report in Venezuela [27]. Biological factor and high cost of these antibiotics could be responsible for the low of resistance for augmentin and vancomycin. An increased resistance of *S. pneumoniae* isolates for amoxicillin (20.4%) and erythromycin (15.0%) were also observed. Our results are similar to those reported in Vilnius [32]. Widely usage of this drug in the clinical practice and its cheap cost could be the cases. Therefore, frequent development of antibiotics resistance can be as a result of increased selection pressure for the resistant strains due to uncontrolled local availability of some antimicrobial agents, leading to frequent use and misuse.

In conclusion, nasopharyngeal carriage of *S. pneumoniae* was common among healthy children in day care centers. Our findings indicated important associations between age, living with younger children, living in a house having a single room, and colonization by *S. pneumoniae* with diminished antimicrobial sensitivity. Therefore, a strict policy with respect to antibiotic prescription together with widespread use of pneumococcal conjugate vaccination could potentially reduce the carriage in the community.

## Limitations

This study carried out in summer and spring only; thus, unable to see the effect of seasonal variation in the colonization in the entire seasons and this could be considered as the limitation of this study. In this study, we did not serotype the pneumococcus, which could have been helpful to look at serotype distribution. Further, this study has not looked at children who were unable to attend day care centers in the study area.

## Abbreviations

AOR: adjusted odd ratio
CI: confidence interval
DCC: day care center
IPD: invasive pneumococcal disease
LRTIs: lower respiratory tract infection
OR: odd ratio
SD: standard deviation
URTIs: upper respiratory tract infection

## References

[1] Vernet G, Saha S, Satzke C, Burgess DH, Alderson M, Maisonneuve JF (2011). Laboratory-based diagnosis of pneumococcal pneumonia: state of the art and unmet needs. Clin Microbiol Infect.

[2] WHO (2014). Pneumonia.

[3] Greenwood B (1999). The epidemiology of pneumococcal infection in children in the developing world. Phil Trans R Soc Lond B.

[4] Usuf E, Bottomley C, Adegbola A, Hall A (2014). Pneumococcal carriage in sub-Saharan Africa: a systematic review. PLOS ONE.

[5] Ministry of Health Federal Republic of Ethiopia. Introducing pneumococcal conjugate vaccine in Ethiopia: Training Manual for Health Workers. 2011.

[6] Backhaus E (2011). Invasive pneumococcal infections.

[7] WHO (2010). Pnemococcal infection.

[8] Ercibengoa M, Arostegi N, Marimón JM, Alonso M, Pérez-Trallero E (2012). Dynamics of pneumococcal nasopharyngeal carriage in healthy children attending a day care center in northern Spain. Influence of detection techniques on the results. BMC Infect Dis.

[9] Ekdahl K (2010). Carriage of pneumococci in healthy children in Gothenburg, Sweden. BMC Infect Dis.

[10] Bogaert D, de Groot  R, Hermans PW (2004). *Streptococcus pneumoniae* colonisation: the key to pneumococcal disease. Lancet Infect Dis.

[11] Otsuka T, Chang B, Shirai T, Iwaya A, Wada A, Yamanaka N, Okazaki M (2013). Individual risk factors associated with nasopharyngeal colonization with *S. pneumoniae* and *Haemophilus influenzae*. Pediatr Infect Dis J.

[12] Scott Giebink G (2001). The prevention of pneumococcal disease in children. N Engl J Med.

[13] Hill PC, Cheung YB, Akisanya A, Sankareh K, Lahai G, Greenwood BM (2008). Nasopharyngeal carriage of *Streptococcus pneumoniae* in Gambian infants: a longitudinal study. Clin Infect Dis.

[14] Givon-Lavi N, Fraser R, Porat N, Dagan R (2002). Spread of *Streptococcus pneumoniae* and antibiotic-resistant *S. Pneumonia* from day-care center attendees to their younger siblings. J Infect Dis.

[15] Mohammed E, Muhe L, Geyid A, Asmelash T, Tessema T, Dejene A (2000). Prevalence of acute respiratory bacterial pathogens in children in Gondar. Ethiop J Health Dev.

[16] Centers for Disease Control and Prevention (2003). Manual for the laboratory identification and antimicrobial susceptibility testing of bacterial pathogens of public health importance in the developing world.

[17] Assefa A, Gelaw B, Shiferaw Y, Tigabu Z (2013). Nasopharyngeal carriage and antimicrobial susceptibility pattern of *S. pneumoniae* among pediatric outpatients at Gondar University Hospital, North West Ethiopia. Pediatr Neonatol.

[18] Leal AL, Castañeda E (1997). Antibiotic susceptibility of *S. pneumoniae* colonizing the nasopharynx of Colombian children with pneumonia. Pan Am J Public Health.

[19] Jette LP, Sinave C (1999). Use of an oxacillin disk screening test for detection of penicillin and ceftriaxone-resistant pneumococci. J Clin Microbiol.

[20] Clinical and Laboratory Standard Institute (2011). Performance standards for antimicrobial susceptibility testing; Twenty-first informational supplement. CLSI document M100eS21.

[21] Espinosa-de los Monteros LE, Jiménez-Rojas V, Aguilar-Ituarte F, Cashat-Cruz M, Reyes-López A, Rodríguez-Suárez R (2007). *Streptococcus pneumoniae* isolates in healthy children attending day-care centers in 12 states in Mexico. Salud Publica Mex.

[22] Granat SM, Mia Z, Ollgren J, Herva E, Das M, Piirainen L, Auranen K, Mäkelä PH (2007). Pneumococcal carriage during the first year of life in Bangladesh. Pediatr Infect Dis J.

[23] Bozio C. Risk factors for bacterial load of pneumococcal colonization among peruvian children. Doctoral Dissertation, Emory University; 2011.

[24] Githii S, Revathi G, Muigai A, Kariuki S (2013). Carriage rate and serotypes of *Streptococcus pneumonia* amongst children in Thika Hospital, Kenya. Afr J Lab Med.

[25] Malfroot A, Verhaegen J, Dubru JM, Van Kerschaver E, Leyman S (2004). A cross-sectional survey of the prevalence of *Streptococcus pneumonia* nasopharyngeal carriage in Belgian infants attending day care centres. Microbiol Infect.

[26] Gary D (2000). Prevention of pneumococcal infections, including the use of pneumococcal conjugate and polysaccharide vaccines and antibiotic prophylaxis. Pediatrics.

[27] Quintero B, Araque M (2006). Serotype profile and antibiotyping of *Streptococcus pneumoniae* strains isolated from nasal carriage in pediatric patients. Invest Clin.

[28] Berezin NE, Cardenuto Leda MD, Ferreira L, Otsuka M (2007). Distribution of *streptococcus pneumonia* serotypes in nasopharyngeal carriage and in invasive pneumococcal disease in sao paulo, brazil. Pediatr Infect Dis J.

[29] Gazi H, Kurutepe S, Surucuoglu S, Teker A, Ozbakkaloglu B (2004). Antimicrobial susceptibility of bacterial pathogens in the oropharynx of healthy school children in Turkey. Indian J Med Res.

[30] Masuda K, Masuda R, Nishi J, Tokuda K, Yoshinaga M, Miyata K (2002). Incidences of nasopharyngeal colonization of respiratory bacterial pathogens in Japanese children attending day-care centers. Pediatr Int.

[31] Vallès X, Flannery B, Roca A, Mandomando I, Sigauque B, Sanz S (2006). Serotype distribution and antibiotic susceptibility of invasive and nasopharyngeal isolates of *Streptococcus pneumoniae* among children in rural Mozambique. Trop Med Int Health.

[32] Staceviciene I, Petraitiene S, Vaiciuniene D, Alasevicius T, Kirsliene J, Usonis V (2016). Antibiotic resistance of *S. pneumoniae*, isolated from nasopharynx of preschool children with acute respiratory tract infection in Lithuania. BMC Infect Dis.

